# Bactericidal efficacy of low dose gaseous ozone against clinically relevant multidrug-resistant bacteria

**DOI:** 10.3389/fmicb.2024.1480433

**Published:** 2024-12-11

**Authors:** Bob Banerjee, Christine Thompson, Victor Nizet, Elisabet Bjånes

**Affiliations:** ^1^Lumos Consulting, Lititz, PA, United States; ^2^Division of Host-Microbe Systems and Therapeutics, Department of Pediatrics, University of California San Diego, La Jolla, CA, United States; ^3^Skaggs School of Pharmacy and Pharmaceutical Sciences, University of California San Diego, La Jolla, CA, United States

**Keywords:** gaseous ozone disinfection, multidrug-resistant bacteria, alternatives to antibiotics, low dose ozone, environmental decontamination

## Abstract

**Introduction:**

Healthcare-associated infections (HAIs) pose a significant challenge in acute care hospitals, particularly in intensive care units, due to persistent environmental contamination despite existing disinfection protocols and manual cleaning methods. Current disinfection methods are labor-intensive and often ineffective against multidrug-resistant (MDR) pathogens, highlighting the need for new, automated, hands-free approaches.

**Methods:**

This study evaluates the bactericidal efficacy of low concentrations of gaseous ozone (5 ppm) against clinically relevant and often MDR bacteria under various concentrations, contact times, temperatures, and environmental conditions.

**Results:**

We observed a 3 log_10_-fold reduction in *Escherichia coli* and *Salmonella Typhimurium* and a 1–2 log_10_-fold reduction in group A *Streptococcus* and methicillin-resistant *Staphylococcus aureus* upon ozone exposure. The bactericidal effect was dose-dependent, with no significant difference between single and repeated exposures. Environmental conditions such as temperature and humidity had minimal impact on low-dose ozone efficacy, with slightly improved bacterial killing at colder temperatures and higher humidity levels. Gaseous ozone also showed significant bactericidal activity against the broad range of Gram-positive and -negative MDR clinical isolates.

**Discussion:**

These findings highlight the potential of low-dose gaseous ozone as a versatile, effective, and hands-free disinfectant for healthcare and other settings. Further research is needed to establish long-term safety and efficacy guidelines for its use in occupied spaces and to explore potential synergy with other contemporary disinfection strategies.

## 1 Introduction

Approximately 75,000 individuals acquire healthcare-associated infections (HAIs) annually in U.S. acute care hospitals (Popovich et al., 2019; Halverson et al., 2022). Environmental contamination significantly contributes to the acquisition of HAIs, particularly in intensive care units (ICUs) (Huang et al., 2006; Drees et al., 2008; Dancer, 2014; Cohen et al., 2018) Many clinically relevant pathogens can survive on inanimate surfaces for extended periods (Porter et al., 2024), and the presence of a previously infected occupant significantly increases the risk of subsequent residents developing HAIs (Mitchell et al., 2023). Current disinfection protocols, which include manual and mechanical cleaning with disinfectants, germicides, and ultrasonic cleaners (Rutala and Weber, 2015; Sheppeard et al., 2022), face challenges due to the development of resistance in many clinical isolates, rendering decontamination both expensive and labor-intensive (Dancer, 2014; Lourbopoulos et al., 2021). Given the persistent issues of understaffing and the growing antibiotic resistance crisis, there is a critical need for new, hands-free disinfection methods (Dancer, 2014).

Ozone (O_3_), a potent oxidant composed of three unstable oxygen atoms, is a naturally occurring, pungent gas found at 10–20 parts per billion (ppb) in the atmosphere and up to 100 ppb in polluted areas. Ozone demonstrates remarkable microbicidal activity by oxidizing bacterial lipids, viral envelopes and capsids, and fungal membranes (Allison et al., 2009; Brié et al., 2018; Xue et al., 2023). Additionally, it generates reactive oxygen species (ROS) that collapse cellular membranes. O_3_ is frequently employed as an environmental decontaminant, particularly in its aqueous form in water treatment plants (Ding et al., 2019; Epelle et al., 2023).

Ozone has also been used therapeutically for skin and oral pathologies for decades (Kim et al., 2009; Liu et al., 2023). Despite these successes, the use of gaseous ozone as an environmental disinfectant has been limited due to its toxicity to humans at levels exceeding 70 ppb. The severity of these effects can be mitigated by using lower doses, shorter durations, and less frequent applications of ozone (Bette et al., 2022; Singh et al., 2023). While high doses of ozone can be harmful, it also offers advantages as a treatment therapy for various human diseases including cancer, bacterial infections, asthma, and viral diseases, due to its disinfectant properties and anti-inflammatory effects (Singh et al., 2023; Elvis and Ekta, 2011; Zanardi et al., 2016). Additionally, ozone has demonstrated effective antimicrobial properties in sustainable food production, wound healing in healthcare, and public environments by penetrating intracellular components of microorganisms and causing oxidative damage (Epelle et al., 2023; Agarwal et al., 2020; Moraes et al., 2021; Premjit et al., 2022; Roth et al., 2023; Neves et al., 2023).

In recent years, interest in low-dose ozone as a cheap and effective environmental disinfectant has grown, particularly in the agriculture and healthcare industries (Shen et al., 2021). Gaseous ozone is a Generally Recognized as Safe (GRAS) agent and is approved by the U.S. Food and Drug Administration (FDA) for use (Bobka, 1993). Several studies have reported conflicting evidence regarding the efficacy of low-dose gaseous ozone against pathogens, with effectiveness against some bacteria but ineffectiveness against COVID-19 (Fontes et al., 2012; Westover et al., 2022; Misawa et al., 2023). Additionally, several studies have assessed ozone’s capability in decontaminating food and bacterial surfaces (Xue et al., 2023; Coll Cárdenas et al., 2011; Cantalejo et al., 2016). Most of these studies utilized a limited number of targets and exposure conditions, making it difficult to generalize the efficacy of low-dose gaseous ozone as a disinfectant.

We aimed to characterize the impact of environmental conditions, including bacterial growth conditions, temperature, and humidity, on the microbicidal capacity of low-dose ozone on clinically relevant bacterial pathogens. We found that ozone was effective at killing both Gram-positive and Gram-negative species in a dose-dependent manner. Additionally, we found that gaseous ozone was equally effective at low and ambient temperatures and was relatively unaffected by humidity. These studies address the current gap in the literature and provide evidence for the use of low-dose gaseous ozone as a potential environmental disinfectant.

## 2 Materials and methods

### 2.1 Bacterial methods

All strains utilized are listed in Table 1. Strains are organized by usage in each figure.

**Table 1:** Bacterial strains.

Figures 1, 2: Strain	Source
Methicillin-resistant *Staphylococcus aureus* TCH1516	Gonzalez et al. (2005)
*E coli* CFT073	Welch et al. (2002)
Group A *Streptococcus* M1T1 5,448	Chatellier et al. (2000)
*Salmonella enterica* serovar Typhimurium	ATCC 14028

Figure 3A: Strain	Source
MRSA JH1	Bosi et al. (2016)
*E. coli ESBL-3*	Clinical isolate, this study
*Pseudomonas aeruginosa* P4	Lin et al. (2015)
*Pseudomonas aeruginosa* PA14	Rahme et al. (1995)
*Acinetobacter baumannii* Lac-4	Harris et al. (2013)
Group A *Streptococcus* NS501 Serotype M14	McKay et al. (2004)
Group B *Streptococcus* 7507-03	Dahesh et al. (2008)
*Serratia marcescens* SR01	Clinical isolate, this study
*Enterococcus faecium* 447	Tran et al. (2013)

Figure 3B: Strain	Source
*E coli* CFT073	Welch et al. (2002)
MRSA Newman Strain	Kuipers et al. (1993)
*Pseudomonas aeruginosa* PA14	Rahme et al. (1995)
Group A *Streptococcus* M1T1 5,448	Chatellier et al. (2000)
*Vibrio cholerae* Inaba El Tor O1 strain C6706	Son et al. (2011)
*Enterococcus faecalis*	ATCC 29212
*Salmonella enterica* serovar Typhimurium	ATCC 14028
*Listeria monocytogenes* 10403S	Datta et al. (2006)
*Lactococcus lactis* NZ9000	Kuipers et al. (1993)
*Bacillus subtilis* 3,610	Liu et al. (2010)
*Pseudomonas fluorescens* Migula	ATCC 13525

#### 2.1.1 Bacterial cultures

*Escherichia coli, Pseudomonas aeruginosa*, *Salmonella Typhimurium* (S. Tm), *Serratia marcescens*, and *Vibrio cholerae* were grown in Luria broth (LB) at 37°C with aeration. *Acinetobacter baumannii* was grown in tryptic soy broth (TSB) at 37°C with aeration. *Listeria monocytogenes* was grown in brain heart infusion broth (BHI) at 37°C with aeration. Methicillin-resistant *Staphylococcus aureus* (MRSA), *Bacillus subtilis*, *Enterococcus faecalis*, and *Lactococcus lactis* were grown in Todd Hewitt Broth (THB) at 37°C with aeration. Group A *Streptococcus* (GAS) and group B *Streptococcus* (GBS) were grown in THB at 37°C without aeration. *Pseudomonas fluorescens* Migula was grown in nutrient broth at 30°C with aeration. For stationary phase cultures, 16–20 h cultures were used except for *P. fluorescens* Migula which was grown for 36 h. For log-phase cultures, stationary phase cultures were diluted 1:20 in fresh media and grown for 2–4 h under appropriate culture conditions until cultures reached mid-logarithmic phase (OD_600_ ~ 0.4).

### 2.2 Gaseous ozone

#### 2.2.1 Gaseous ozone chamber

We constructed a 28-liter^3^ ozone chamber for this study, capable of controlling both ozone and humidity levels. Ozone was produced by an ozone generator (Model: 1000 mg/h; Ambohr Electric Limited, Fengdong New Town, Xi’an, Shaanxi, China), and vented into a humidity- controlled chamber. Ozone concentration was measured using an ozone sensor (Model:110-4xx, Interlink Electronics, Irvine California) and flow rate (0.1–5 ppm) was adjusted to maintain the desired concentration. We built an ozone destructor (MINSLITE-B, Hunan, China) that decomposed residual ozone immediately after reaching the desired contact time. Humidity was measured using a humidity and temperature sensor (Model: SHT-31, Sensiron AG, Stäfa Switzerland) and the humidity was adjusted with molecular grade H_2_O. Ambient humidity (~55–70%) was used unless otherwise indicated.

#### 2.2.2 Refrigerated ozone chamber

Ozone was produced by an ozone generator (Model: 1000 mg/h; Ambohr Electric Limited, Fengdong New Town, Xi’an, Shaanxi, China) placed inside a compact refrigerator (74 liters^3^, Walmart). Ozone concentration was measured using an ozone sensor (Model ZE14-O3, Winsen Electronics Technology Co., Ltd., Zhengzhou, China) and flow rate (0.1–5 ppm) was adjusted to maintain the indicated concentration.

#### 2.2.3 Gaseous ozone treatment

Note: to compare gaseous ozone to other published studies, ozone concentration can be expressed in multiple ways. We report concentrations in volumetric ppm O_3_ and contact time (CT) = ppm x exposure time. Equivalent measures are listed in Table 2. Additional ozone calculation information can be found at Oxidation Technologies (Ozone Equipment Manufacturer and Ozone System Integrators, 2024).

**Table 2:** Gaseous ozone calculation.

Measure	Calculation	Equivalent to 1 volumetric ppm O_3_
Contact time	1 PPM × Minute = 1 ppm O_3_ × 1 min = 1 CT	1 CT
Volume	1 mg/L = 1 g/m^3^ = 1 ug/ml = 467 ppm O_3_	2.14 mg O_3_/m
Weight in air	1% O_3_ = 12.8 g/m^3^ = 7,284 ppm O_3_	0.00014% O_3_

Ozone concentration can be reported in multiple ways. Above table lists calculations for reporting gaseous ozone concentration and equivalent measures to 1 volumetric ppm O_3_.

Unless otherwise indicated, stationary-phase cultures were serially diluted in PBS and spot-plated onto appropriate agar. Petri dishes were placed in the chamber at the indicated ozone contact times and humidities with the lid off, exposing the surface for the duration of the CT. For humidity experiments, control plates were placed in the chamber for the same amount of time with the tested humidity in the absence of ozone. For refrigerated experiments, control plates were placed in the refrigerated chamber for the same duration without ozone exposure to account for the impact of temperature on bacterial growth inhibition.

##### 2.2.3.1 Dose response experiments

Stationary-phase *E. coli, S*. Tm, GAS, and MRSA were serially diluted in PBS in triplicate, plated on Luria agar (LA) or Todd Hewitt Agar (THA) plates, and exposed to 5 ppm O_3_ for 0, 25, 50, 100,200, or 400 O_3_ CT.

##### 2.2.3.2 Repeated exposure experiments

Stationary-phase *E. coli, S*. Tm, GAS, and MRSA were serially diluted in PBS in triplicate, plated on LA or THA plates, and exposed to a single dose of 5 ppm O_3_ for 360 CT or three doses of 5 ppm O_3_ 200 CT with 30–60 min recovery time at normal O_2_ concentrations between exposures. Control plates were left untreated at ambient O_2_.

##### 2.2.3.3 Logarithmic vs. stationary phase experiments

*E. coli, S*. Tm, GAS, and MRSA in stationary or mid-log phase (OD_600_ 0.4–0.6) were serially diluted in PBS in triplicate, plated on Luria agar (LA) or Todd Hewitt agar (THA) plates and exposed to 0, 25, 100, or 400 O_3_ CT.

##### 2.2.3.4 Refrigerated exposure experiments

Stationary phase *E. coli, S*. Tm, GAS, and MRSA were serially diluted in PBS in triplicate, plated on LA or THA plates and exposed to 5 ppm O_3_ for 0, 25, 100, or 400 O_3_ CT at 4°C or room temperature (RT, 21–23°C). For extended refrigerated exposures, plates were exposed to 1 ppm O_3_ for 0 or 360 O_2_ CT. Untreated plates were incubated in the absence of ozone at 4°C or RT for the equivalent exposure time. All plates were then incubated overnight at 37°C.

##### 2.2.3.5 Humidity exposure experiments

Stationary phase *E. coli, S*. Tm, GAS, and MRSA were serially diluted in PBS in triplicate, plated on LA or THA plates and exposed to 5 ppm O_3_ 200 CT at low (<40%), ambient (40–60%), and high (>70%) humidity.

##### 2.2.3.6 Clinical isolate experiments

Stationary phase MRSA, *E. coli*, *P. aeruginosa*, GAS, GBS, *S. marcescens, E. faecium, V. cholerae, Salmonella, L. monocytogenes, L. lactis, B. subtilis, P. fluorescens* and *A. baumannii* were grown in appropriate media, serially diluted in PBS in triplicate, and plated on appropriate agar as described above. Plates were exposed to 5 ppm O_3_ for a 400 CT or 1 ppm O_3_ 360CT. Control plates were left untreated at ambient O_2_. All plates were then incubated overnight at 37°C except *P. fluorescens* which was incubated at 30°C.

##### 2.2.3.7 Fitness studio pilot experiment

An ozone generator was placed inside a fitness studio, and the room was exposed to 0.5 ppm for 1 h for three consecutive evenings. Six sites were swabbed with pre-moistened sterile cotton swabs for microbial growth prior to exposure and after the final exposure. Swabs were vigorously inoculated into Letheen broth (3M) to transfer microbes, TSA was serially diluted, plated in triplicate on TSA plates, and grown at 37°C overnight for enumeration.

##### 2.2.3.8 Data analysis

Statistical analysis was performed using GraphPad Prism v10. Comparisons between two groups were conducted using a two-tailed Student’s *t*-test, while comparisons among three or more groups were conducted using a one-way analysis of variance (ANOVA). Unless otherwise indicated in the figure legends, graphs display the means of technical replicates from three or more independent biological replicates ± standard error of the mean (SEM). *p* values <0.05 were considered statistically significant.

## 3 Results

**Figure 1:**
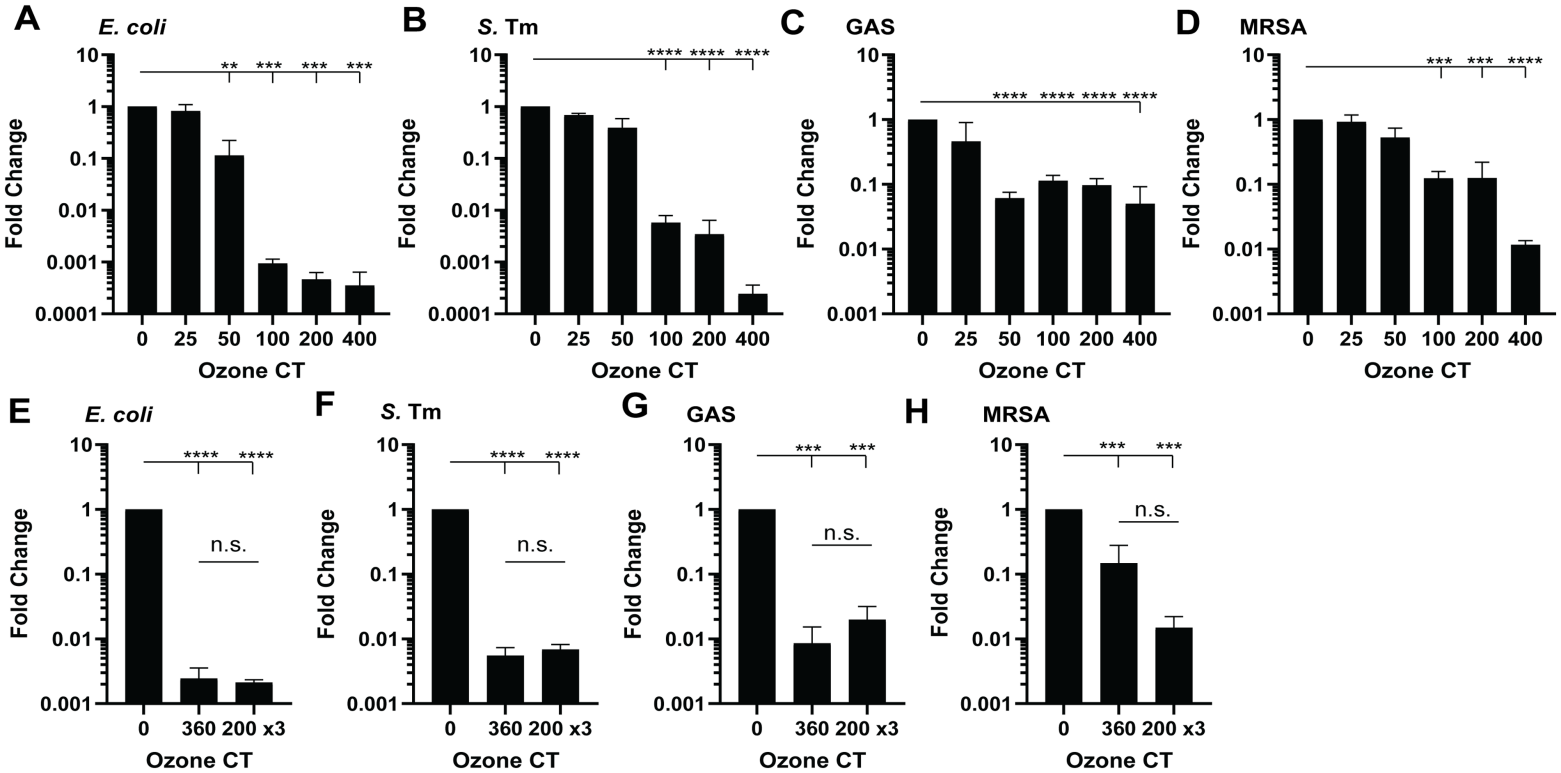
Gaseous ozone is effective against Gram-negative and Gram-positive bacteria in a dose-dependent manner. **(A–D)** Fold change in colony forming units (CFUs) from stationary phase **(A)** *E. coli*, **(B)** *Salmonella enterica* serovar Typhimurium (S. Tm), **(C)** Group A *Streptococcus* (GAS), and **(D)** methicillin-resistant *Staphylococcus aureus* (MRSA) treated with 5 ppm gaseous O_3_ to 25, 100, 200, and 400 CT. **(E–H)** Fold change in CFUs of **(E)** *E. coli*, **(F)** *S*. Tm, **(G)** GAS, and **(H)** MRSA treated with 5 ppm to 360 CT once or 200 CT three times with 30–60 min rest between each cycle. Pooled means ± SEM from three independent experiments. n.s., not significant. * *p* < 0.05, ** *p* < 0.01, *** *p* < 0.001, **** *p* < 0.0001.

We first tested the microbicidal activity of gaseous ozone, selecting *E. coli* and *S. typhimurium* as representative Gram-negative strains, and GAS and MRSA as representative Gram-positive strains. Strains were exposed to gaseous ozone at 5 ppm for varying CT. We observed a 3 log_10_-fold reduction in both *E. coli* and *S. typhimurium* after exposure compared to controls (Figures 1A,B). GAS and MRSA showed a 1–2 log_10_-fold reduction after exposure to increasing concentrations of O_3_ (Figures 1C,D), indicating that gaseous ozone kills in a dose-dependent manner. To assess whether repeated exposure enhances killing, we exposed strains to a single 360 CT dose or three doses of 200 CT with recovery periods between each dose. We found no significant difference in bacterial survival between single and multiple exposures, suggesting that a single potent dose is sufficient to reduce bacterial survival (Figures 1E–H).

Strains were exposed to gaseous ozone at 5 ppm for varying contact times (CT). We observed a three log_10_-fold reduction in both *E. coli* and *S. typhimurium* after exposure compared to controls (Figures 1A,B). GAS and MRSA showed a 1–2 log_10_-fold reduction after exposure to increasing concentrations of O_3_ (Figures 1C,D), indicating that gaseous ozone kills in a dose-dependent manner. To assess whether repeated exposure enhances killing, we exposed strains to a single 360 CT dose or three doses of 200 CT with recovery periods between each dose. We found no significant difference in bacterial survival between single and multiple exposures, suggesting that a single potent dose is sufficient to reduce bacterial survival (Figures 1E–H).

**Figure 2:**
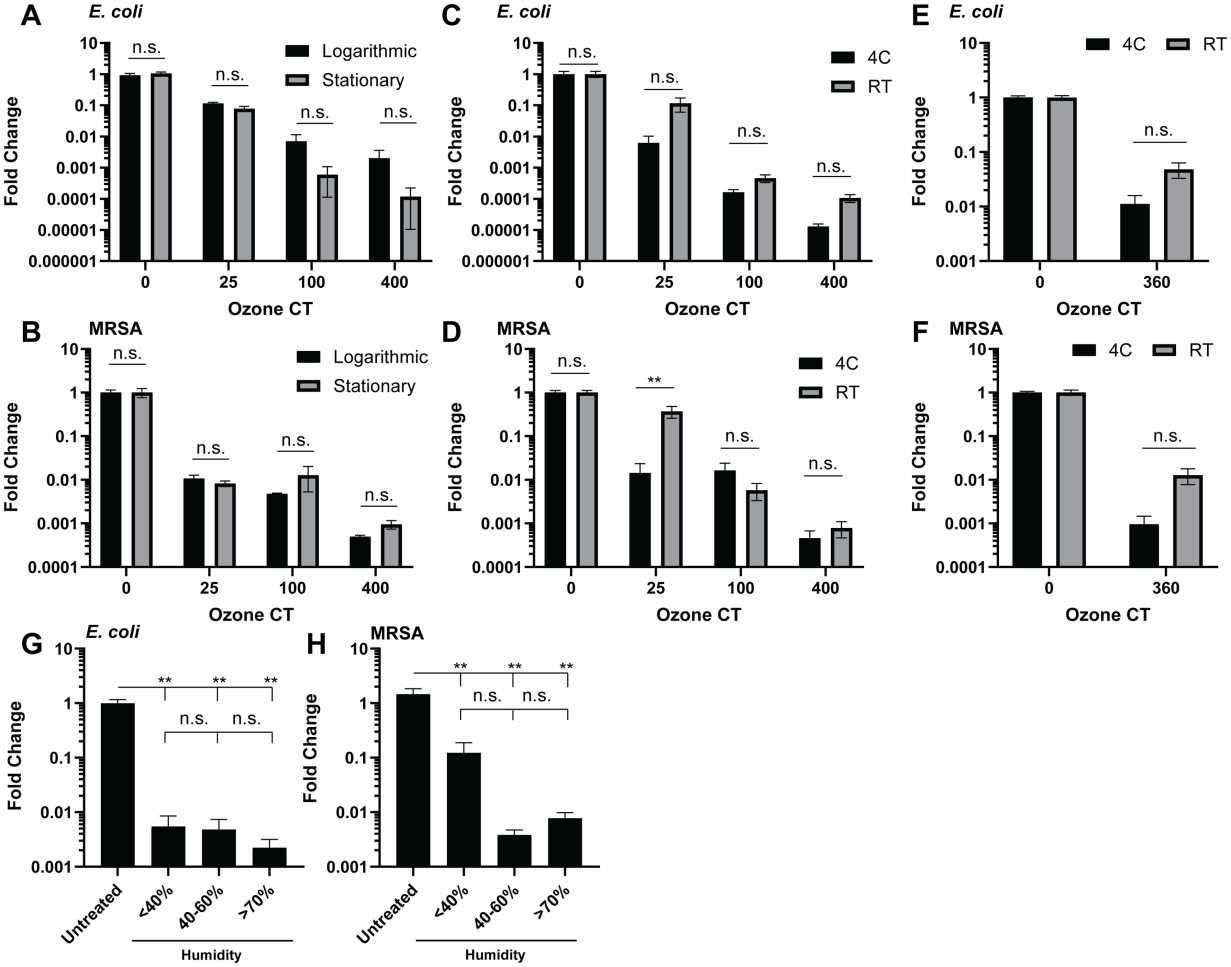
Ozone killing efficacy is largely indifferent to microbial growing conditions, ambient temperature, and humidity. **(A,B)** CFUs of **(A)** *E. coli* and **(B)** MRSA grown at stationary and logarithmic phase treated with 5 ppm O_3_ to 200 CT. **(C–F)** Colony forming units (CFUs) from stationary phase *E. coli* and MRSA treated with **(C,D)** 5 ppm O_3_ to 0, 25, 100, and 400 CT at 4°C or room temperature (RT) or **(E,F)** 1 ppm O_3_ 360 CT at 4C and RT. **(G,H)** CFUs of **(G)** *E. coli* and **(H)** MRSA treated with 5 ppm O_3_ to 200 CT at low (<40%), Ambient (40–60%), or high (>70%) humidity. **(A,B)** Representative of two independent experiments. **(C–F)** three experiments pooled. n.s., not significant. * *p* < 0.05, ** *p* < 0.01, *** *p* < 0.001, **** *p* < 0.0001.

Next, we tested the impact of environmental conditions on the bactericidal capacity of gaseous ozone by exposing bacteria grown in logarithmic and stationary phases to O_3_. We did not find consistent differences in killing between growth phases of *E. coli* and MRSA, although stationary-phase MRSA was modestly, though not significantly, more resistant to killing (Figures 2A,B). Given the significant interest in applying ozone to refrigerated food products, we examined the impact of temperature on gaseous ozone efficacy by exposing *E. coli* and MRSA to gaseous O_3_ at ambient temperature (~21–22°C) and at 4°C. Gaseous ozone was slightly more effective at killing MRSA and *E. coli* at colder temperatures, though the difference did not achieve statistical significance (Figures 2C–F). We also investigated the impact of humidity on ozone’s killing capacity by testing low humidity (<40%), ambient humidity (40–60%), and high humidity (>70%). Different humidity levels did not impact the ability of ozone to kill *E. coli* (Figure 2G). There was a modest, though not statistically significant, improvement in the bactericidal activity of gaseous O_3_ against MRSA at higher humidity levels (Figure 2H). Collectively, these results indicate that gaseous ozone is effective against bacteria in both quiescent and active growth phases and that its killing capacity is relatively unaffected by low temperatures and varying humidity levels.

**Figure 3:**
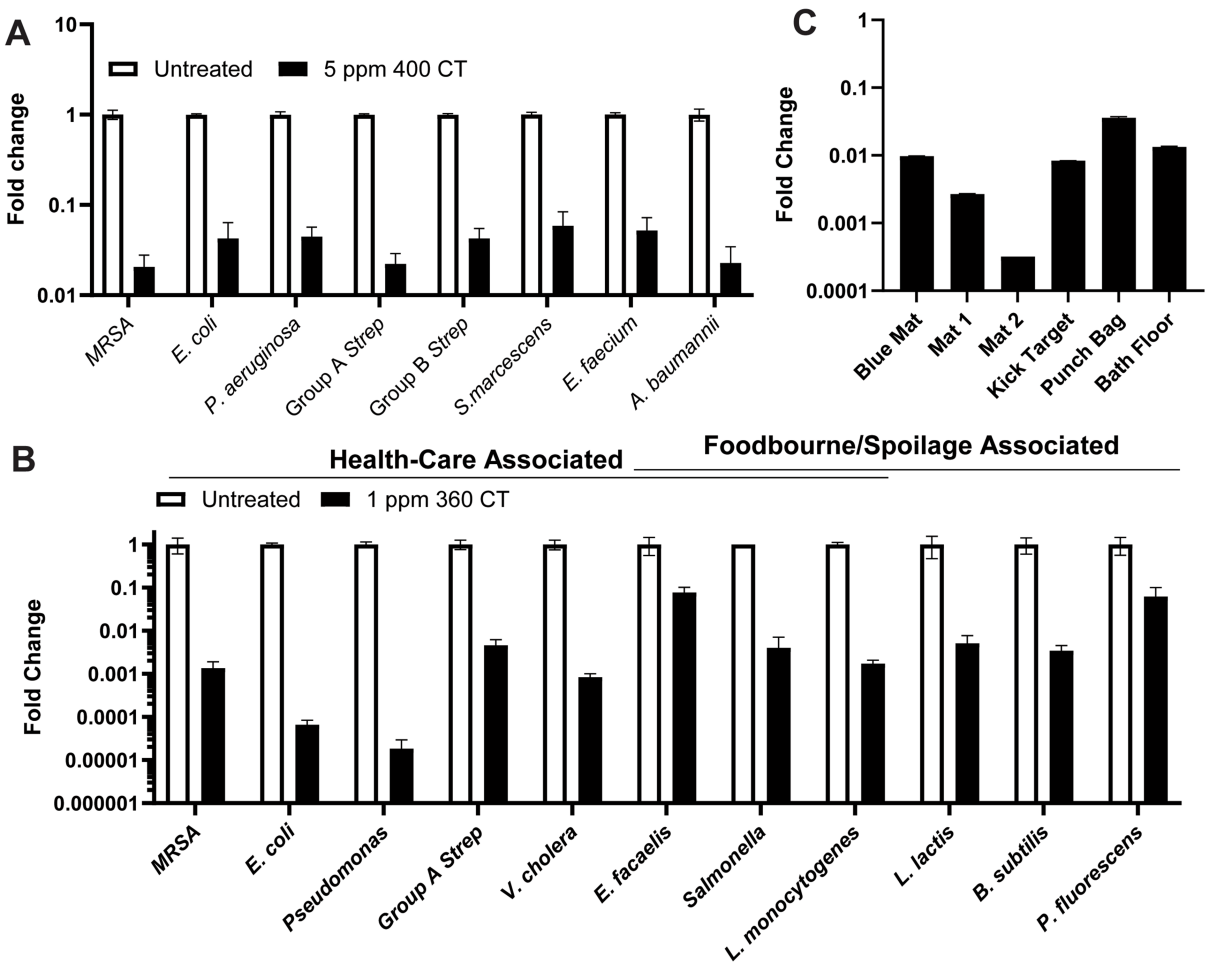
Gaseous ozone is effective against clinically relevant bacterial pathogens. **(A)** CFUs from clinical isolates treated with 5 ppm 400 CT. **(B)** CFUs from healthcare associated and foodborne/spoilage associated bacteria treated with 1 ppm O_3_ 360 CT. Healthcare associated pathogens include MRSA, *E. coli*, *Pseudomonas aeruginosa*, GAS, *Vibrio cholerae*, *Enterococcus faecalis*, *S*. Tm, and *Listeria monocytogenes*. Foodborne pathogens include *E. faecalis, S*. Tm, *L. mono*, *Lactococcus lactis*, *Bacillus subtilis*, and *Pseudomonas fluorescens*. Pooled means ± SEM from two-five independent experiments. **(C)** Fold change in CFUs from six sampled sites at a yoga studio after exposure to 0.5 ppm for 1 h on three consecutive nights over a weekend in a pilot application study. n.s., not significant. * *p* < 0.05, ** *p* < 0.01, *** *p* < 0.001, **** *p* < 0.0001.

Lastly, we examined the ability of ozone to kill a panel of clinical isolates of important human pathogens. We exposed strains of *A. baumannii*, MRSA, *E. coli*, *P. aeruginosa*, GAS, GBS, *E. faecium*, and *S. marcescens* to a single dose of gaseous ozone and found significant bactericidal activity against all strains tested (Figure 3A). We further tested a broader panel of isolates relevant to both human health and the food industry. Consistent with previous findings, a single low-dose exposure of gaseous ozone effectively reduced microbial counts by at least 10-fold (Figure 3B). As a proof of principle, we conducted a pilot study where an ozone generator was placed in a fitness studio over a weekend. Six sites were sampled for microbial counts before and after exposure to gaseous ozone at 0.5 ppm for 1 h over three consecutive nights. All six sites showed reduced bacterial counts after O_3_ exposure (Figure 3C). These findings collectively demonstrate the potential of gaseous ozone as a hands-free microbicide effective against MDR bacterial species.

## 4 Discussion

The findings from our study demonstrate the robust bactericidal efficacy of low-dose gaseous ozone against a variety of MDR, clinically relevant pathogens, highlighting its potential as a hands-free environmental disinfectant. Our results showed that a single potent dose of ozone was sufficient to achieve significant reductions in bacterial counts. The lack of enhanced killing with repeated exposures suggests that ozone’s bactericidal effect is dose-dependent rather than frequency-dependent.

Ozone’s microbial inactivation stems from its ability to induce microorganisms such as bacteria, fungi, and mold, to generate reactive oxygen species (ROS), which then attack their cell membranes, particularly the polyunsaturated fatty acids, leading to lipid peroxidation and microbial inactivation (Brié et al., 2018; Xue et al., 2023; Epelle et al., 2023; Elvis and Ekta, 2011; Agarwal et al., 2020; Moraes et al., 2021; Premjit et al., 2022; Roth et al., 2023; Neves et al., 2023; Pagès et al., 2020; Rangel et al., 2021). This effect is more evident in humidified ozone applications, where spore swelling occurs more severely, enhancing the passage of ROS and resulting in the collapse or rupture of the cell membrane (Epelle et al., 2023; Agarwal et al., 2020; Moraes et al., 2021; Premjit et al., 2022; Roth et al., 2023; Neves et al., 2023; Bhilwadikar et al., 2019; Tizaoui et al., 2022). When ozone is used against viruses, it damages their lipid envelopes and protein capsids, rendering them unable to infect hosts (Allison et al., 2009). This damage extends to the genome and RNA, impairing the virus’s ability to reproduce. Furthermore, the reaction with ROS can produce secondary reactive species, which can intensify the inactivation process (Farooq and Tizaoui, 2023).

A critical aspect of our study was evaluating the impact of environmental conditions on ozone’s efficacy. Previous studies have found mixed effects of microclimate on O_3_ (Pironti et al., 2021; Blanco et al., 2021; Grignani et al., 2020; Hudson et al., 2009). Our findings indicate that gaseous ozone maintains its bactericidal properties across various temperatures and humidity levels. Specifically, ozone was slightly more effective at colder temperatures (~4°C) compared to ambient temperature (~21–22°C), and higher humidity levels modestly improved killing, particularly for MRSA, though these differences were not statistically significant. Our findings are consistent with those of De Caro et al., which showed mild impacts of humidity and temperature on the bactericidal activity of O_3_ against *E. coli* (Pironti et al., 2021). Furthermore, we observed a slight but nonsignificant increase in microbicidal efficacy at higher humidity levels compared to low humidity, aligning with previous reports demonstrating maximal antiviral O_3_ activity against COVID-19 at high humidity (Hudson et al., 2009). Collectively, these results suggest that ozone can be an effective disinfectant in diverse environmental settings, including refrigerated environments. This is particularly relevant to the food industry, which continually combats mold and bacterial growth in walk-in refrigerators and refrigerated trucks where food is stored and transported. Regular low-dose gaseous ozone application to these spaces could reduce microbial contamination, prevent food spoilage, and result in cost savings.

The effectiveness of ozone against a broad range of clinical isolates, including *A. baumannii*, *P. aeruginosa*, and *S. marcescens*, underscores its potential application in healthcare settings to combat healthcare-associated infections (HAIs). The growing threat of MDR infections is difficult to overstate, with projections estimating 10 million deaths annually by 2050 if infection trends continue unchecked (O’Neil, 2014). Of the 22 new antibiotics approved between 2012 and 2022, only two were considered first-in-class, or novel, antibiotics (García-Castro et al., 2023), and five are no longer available (McKenna, 2020). Despite standardized cleaning protocols, a previously room occupancy by infected patients remains a major risk factor for HAIs due to the ability of MDR pathogens to leverage virulence factors that facilitate their persistence in highly inhospitable healthcare environments (Mitchell et al., 2023). Given the dwindling supply of antibiotics and limitations of current disinfection methods, ozone offers a promising alternative that could be integrated into infection control protocols in a cost-effective manner.

While gaseous O_3_ cannot completely replace manual cleaning, we envision its use in conjunction with current treatments to reduce the risk of HAIs and eliminate environmental reservoirs of nosocomial pathogens. O_3_ could be particularly useful in disinfecting hard-to-reach spaces or areas where manual cleaning is impractical. As a proof of concept, we conducted a pilot study in which an ozone generator was placed in a fitness studio. Three one-hour exposures to 0.5 ppm gaseous O_3_ on successive evenings reduced microbial counts by 2–3 log_10_-fold across multiple surfaces. These findings support the practical application of ozone and suggest it could be implemented in clinics, schools, and other high-risk settings.

While our study provides compelling evidence for the use of low-dose gaseous ozone as an environmental disinfectant, it also highlights areas for future research. Long-term studies assessing the safety and efficacy of continuous ozone use in occupied spaces are necessary to establish implementation guidelines. Additionally, exploring the synergistic effects of ozone with other disinfection methods could further extend its utility in various settings.

## Data Availability

The raw data supporting the conclusions of this article will be made available by the authors, without undue reservation.
